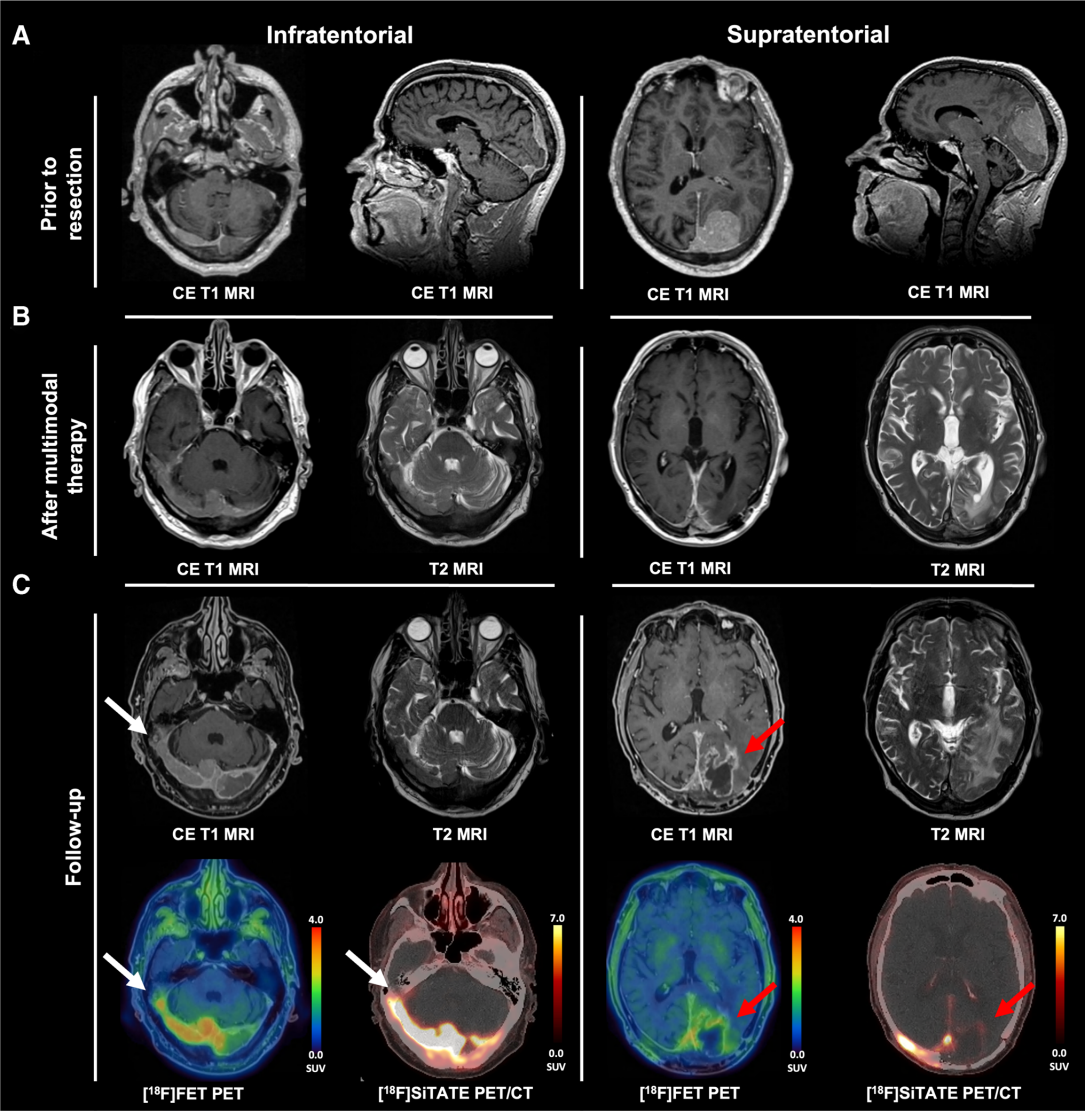# The value of [^18^F]FET PET and somatostatin receptor imaging for differentiating pseudoprogression in residual meningioma

**DOI:** 10.1007/s00259-023-06479-8

**Published:** 2023-10-28

**Authors:** Katharina J. Müller, Annamaria Biczok, Christian Schichor, Louisa von Baumgarten, Nathalie L. Albert

**Affiliations:** 1 Department of Neurology, University Hospital Munich, Ludwig-Maximilians-Universität München, Campus Grosshadern, Marchioninistr. 15, 81377 Munich, Germany; 2 Department of Neurosurgery, University Hospital Munich, Ludwig-Maximilians-Universität München, Campus Grosshadern, Marchioninistr. 15, 81377 Munich, Germany; 3 Department of Nuclear Medicine, University Hospital Munich, Ludwig-Maximilians-Universität München, Campus Grosshadern, Marchioninistr. 15, 81377 Munich, Germany

## Image of the month

An 84-year-old male presented with transitional meningioma WHO° 1 (Figure part A, prior to resection). After completed therapy (resection, cyberknife radiosurgery, fractionated radiation), he showed right-sided residues infiltrating the transverse and sigmoid sinuses (Figure part B, after multimodal therapy). At follow-up 12 months later, MRI showed a new heterogeneous contrast enhancement in the left occipital resection cavity, suggestive of tumor progression (Figure part C, follow-up) [1]. Due to limited availability of somatostatin receptor (SSTR) PET imaging, [^18^F]FET PET was performed as an alternative method. The left occipital lesion showed minor radionuclide uptake on [^18^F]FET PET (TBR_max_: 2.4, TBR_mean_: 1.2; red arrow), whereas the right-sided meningioma showed intense uptake (TBR_max_: 4.6; white arrow). Analysis of [^18^F]FET uptake dynamics revealed decreasing time–activity curves (TTP_min_:12.5 min) in the right-sided meningioma and increasing curves in the left occipital lesion. Three weeks later, we performed SSTR imaging using [^18^F]SiTATE, showing typical SSTR expression of the right-sided meningioma (SUV_max_: 17.1; white arrow), but no typical SSTR expression in the left occipital lesion (SUV_max_: 1.9; red arrow) [2]. Together with the moderate [^18^F]FET uptake, these findings were interpreted as pseudoprogression, confirmed by further follow-up.

The incidence of posttherapeutic pseudoprogression in meningioma is still unknown but considered rare [3]. With an increasing range of treatment options, diagnostic strategies are required to distinguish tumor recurrence more accurately from pseudoprogression [3]. However, when rapid clinical access to SSTR imaging is limited, this may delay diagnosis [4, 5]. To our knowledge, this is the first case demonstrating the value of dual tracer PET imaging in the detection of pseudoprogression in meningioma.